# Mastering your fellowship: Part 3, 2023

**DOI:** 10.4102/safp.v65i1.5728

**Published:** 2023-05-16

**Authors:** Klaus B. von Pressentin, Mergan Naidoo, Arun Nair, Ts’epo Motsohi, Tasleem Ras

**Affiliations:** 1 Division of Family Medicine, Department of Family, Community and Emergency Care, Faculty of Health Sciences, University of Cape Town, Cape Town, South Africa; 2 Department of Family Medicine, University of KwaZulu-Natal, Durban, South Africa; 3 Department of Family Medicine, University of the Free State, Kimberley, South Africa; 4 Robert Magaliso Sobukwe Hospital, Northern Cape Department of Health, Kimberley, South Africa; 5 Division of Family Medicine and Primary Care, Department of Family and Emergency Medicine, Faculty of Medicine and Health Sciences, Stellenbosch University, Cape Town, South Africa

**Keywords:** family physicians, FCFP (SA) examination, family medicine registrars, postgraduate training, national exit examination, eye health

## Abstract

The series, ‘Mastering your Fellowship’, provides examples of the question formats encountered in the written and clinical examinations, Part A of the Fellowship of the College of Family Physicians of South Africa (FCFP [SA]) examination. The series is aimed at helping family medicine registrars (and their supervisors) in preparing for this examination.

## Introduction

This section in the *South African Family Practice* journal is aimed at helping registrars in preparing for the Fellowship of the College of Family Physicians of South Africa (FCFP [SA]) Final Part A examination and will provide examples of the question formats encountered in the written examination: multiple choice question (MCQ) in the form of single best answer (SBA – Type A) and extended matching question (EMQ – Type R); short answer question (SAQ), questions based on the Critical Reading of a Journal article (CRJ: evidence-based medicine) and an example of an objectively structured clinical examination (OSCE) question. Each of these question types is presented based on the College of Family Physicians blueprint and the key learning outcomes of the FCFP (SA) programme. The MCQs are based on the 10 clinical domains of family medicine, the SAQs are aligned with the five national unit standards and the critical reading section will include evidence-based medicine and primary care research methods.

This month’s edition is based on unit standard one (Effectively manage themselves, their team and their practice, in any sector, with visionary leadership and self-awareness, to ensure the provision of high-quality, evidence-based care), unit standard two (Evaluate and manage patients with both undifferentiated and more specific problems cost-effectively according to the bio-psycho-social approach), unit standard three (Improve the health and quality of life of the community) and unit standard five (Conduct all aspects of healthcare in an ethical and professional manner). The clinical domain covered in this edition is eye health. We suggest that you attempt to answer the questions (by yourself or with peers or supervisors), before finding the model answers online: http://www.safpj.co.za/.

Please visit the Colleges of Medicine website for guidelines on the Fellowship examination: https://www.cmsa.co.za/view_exam.aspx?QualificationID=9.

We are keen to hear about how this series is assisting registrars and their supervisors in preparing for the FCFP (SA) examination. Please email (editor@safpj.co.za) us with your feedback and suggestions.

## Multiple choice question (MCQ): Single best answer

**FIGURE 1:**
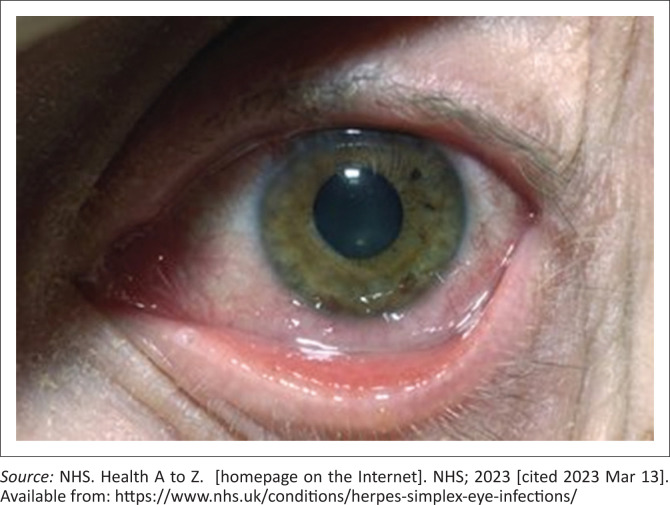
Multiple choice question clinical scenario.

A 65-year-old male presented with a red eye associated with a clear watery discharge, itchiness and pain for the last 2 days. Only one eye is affected. He reports mild blurring of vision. An image of the eye is shown in Figure 1. What would you prescribe as the most appropriate next step?

- Acyclovir ophthalmic ointment
- Chloromycetin ophthalmic ointment
- Oxymetazoline eye drops
- Reassurance and advice on cold compressors
- Steroid eye drops

Answer: (*a*)

### Discussion

The red eye is a frequent presentation to primary care, so it is essential to distinguish potentially serious eye conditions from less severe causes of acute red eye. Common causes include conjunctivitis, keratitis, uveitis, episcleritis, scleritis, acute angle closure glaucoma, a foreign body and subconjunctival haemorrhage. In assessing the red eye, one needs to elucidate if the following symptoms and signs are present:

- Is there pain and irritation?
- Is there a discharge? If yes, describe the type of discharge. Also, assess whether the discharge is present unilateral or bilateral.
- Is photophobia present?
- Is there associated visual loss?
- What is the severity of the red eye?
- Are there pupillary changes?
- Describe the distribution of redness. Is it diffuse, localised, or is it a ciliary flush?

Conjunctivitis is an inflammation of the conjunctiva that is usually bilateral, has variable pain and irritation and often has diffuse redness. The discharge is common and can be watery, mucoid, purulent or mucopurulent. Allergic conjunctivitis may be related to seasonal changes, that is, spring catarrh. The discharge is often mucoid or watery. There may be palpebral follicles or papillae present. The inflammation is prominently perilimbal. Treatment is with antihistamine, cromoglycate or steroid drops if very severe.

Viral conjunctivitis is often associated with a profuse watery discharge and is very common. Keratitis and tarsal follicles may be present. It may be associated with pre-auricular lymphadenopathy. Adenovirus infection may present with epidemic keratoconjunctivitis and pharyngo-conjunctival fever. Treatment for this is supportive management. A more severe form of viral conjunctivitis is because of the herpes simplex virus. Infection is often because of primary infection. Infection is usually unilateral and is associated with pain and blurring of vision. If such symptoms are present, fluorescein staining is advised. Illuminate the cornea with an ophthalmoscope with a cobalt blue filter, with the brightest setting. Punctate stains or linear branching (dendritic) ulcers may be seen in herpes simplex keratoconjunctivitis. A referral is advised if the corneal lesion is not clean or clear, or has whitish areas within the epithelial scar bed; if the corneal staining area is not smaller within 24 h; if there is a history of recurrence; and, if you are worried about disciform keratitis.

Bacterial conjunctivitis is associated with a mucopurulent discharge and may be because of *Staphylococcus epidermidis, Staphylococcus aureus, Haemophilus influenzae, Streptococcus* spp. and *Pseudomonas aeruginosa* (contact lens wearers) and *Neisseria gonorrhoea*. The treatment involved the frequent instillation of antibiotic drops, for example, chloramphenicol. For *N. gonorrhoea*, intramuscular ceftriaxone is also prescribed. Chlamydial infection causes adult inclusion conjunctivitis and is because of serotypes D to K. This infection presents with conjunctival follicles and lid swelling. Treatment is with topical and systemic tetracycline.

The unilateral nature of the case presented with the blurring of vision should alert the clinician of the possibility of herpes conjunctivitis and keratitis. Then fluorescein staining and prompt initiation of the definite treatment are mandatory.

**Further reading**

- Pons J. Chapter 80: How to examine the eye. In: Mash B, Brits H, Naidoo M, Ras T, editors. South African family practice manual. 4th ed. Braamfontein: Van Schaik; 2023.
- Pons J. Chapter 81: How to treat the eye. In: Mash B, Brits H, Naidoo M, Ras T, editors. South African family practice manual. 4th ed. Braamfontein: Van Schaik; 2023.
- South African Department of Health. Hospital-level standard treatment guidelines and rssential medicines list. Pretoria: South African National Department of Health; 2019.
- Albrecht S. Conjunctivitis [homepage on the Internet]. New York, NY: Medscape; 2011 [cited 2023 Jan 23]. Available from: https://www.medscape.com/viewarticle/741938_1

## Short answer question (SAQ): The family physician’s role as a champion of community-orientated primary care in the clinical domain of ophthalmology

*This question was previously used in a FCFP(SA) written article.*

You are the family physician working in a community health centre (CHC) in South Africa. Disability because of cataracts seems to be increasing in the community. The nursing staff suggest that the CHC assists the community in addressing this disability.

1. List five questions you would ask to decide if this is a priority in the community. (5 marks)
2. How would you go about establishing answers to three of the questions that you listed in question 1 above? (3 marks)
3. If you decide that visual loss is a priority in this community, what primary and secondary prevention strategies could you implement in the community? (5 marks)
4. If you decide that visual loss is a priority in this community, what facility-based primary and secondary prevention interventions could you implement? (5 marks)
5. Who could you approach as potential partners in addressing this disability burden for the community? (2 marks)

Total: 20 marks

Model answers

1. **List five questions you would ask to decide if this is a priority in the community. (5 marks)**

(*The approach to this question requires the answer in the following points for prioritisation as per the Family Practice Manual*)

- How *common* is the problem of cataracts?
- How *serious* is the problem?
- To what extent is the community *concerned* about it?
- Is it *feasible* to intervene?
- Will an intervention be *effective*?

2. **How would you go about establishing answers to three of the questions that you listed in question 1 above? (3 marks)**

*One point for one mark under any of the three questions chosen as listed below:*

- How common is the problem of cataracts?:
  ■ Suggest a measure of prevalence such as the percentage of people seen at the CHC diagnosed with cataracts in the last year. Establish how many of those diagnosed with cataracts also have a significant visual disability as per visual acuity scores (and possibly the reasons given for cataracts).
  ■ Use ward-based primary health care (PHC) outreach teams while doing their house visits to identify those with vision loss that have not yet sought help at the CHC.
  ■ Start looking at incidence: the number of new cases developing cataracts per year.
- How serious is the problem? (Weighed against other health issues faced by the community):
  ■ Impact of the disability on the community and families if no intervention is taken now.
  ■ Backlog: The number of cataract cases identified and not operated on at present in the community (Backlog + received surgery = burden of cataract).
- To what extent is the community concerned about it?:
  ■ Does the community feel the same concern the nursing staff have observed? Explore what the community feels, thinks and does regarding this problem and if they see it as a priority area for intervention. Discuss with community elders, leaders, and community health workers (CHWs). Assess and identify to address the barriers (distance to hospital, fear of surgery, long waiting list, insufficient manpower, materials) to cataract surgery with the community.
- Is it feasible to intervene?:
  ■ Is it possible to come up with interventions as a multidisciplinary team with the community, health facilities, ophthalmologists, optometrists and outside partners (alternative healers or traditional healers) to address the issue of cataracts? Do the community leaders buy into the proposed intervention plans?
- Will an intervention be effective?:
  ■ Can the CHC be able to come up with realistic strategies with the resources available locally, with community support and help from networking with other resources available? Discuss strategies for monitoring if the intervention is working in the future and establish a cost-effective plan that is agreeable to all the stakeholders.
  ■ Identify modifiable risk factors associated with cataracts among the patients seen in the CHC (diabetes, smoking, chronic steroid use, chronic ultraviolet exposure). Train staff to screen such patients for cataracts.

3. **If you decide that visual loss is a priority in this community, what primary and secondary prevention strategies could you implement in the community? (5 marks)**

*One mark for any of the five points given below*

Health education in the community may consist of primary prevention (preventing cataracts from forming) and secondary prevention (preventing progression to cataract blindness in patients with cataracts):

- Motivate people to have regular eye checks after the age of 40 years and seek help with changes in vision at the local CHC.
- Monitor the changes in vision in family members by other family members (e.g. change in baseline while reading, watching television, etc.) and seek help early.
- Health education strategies in the community should be orientated to modify risk factors for conditions associated with visual loss. This can be in partnership with any other services providing eye care in the community such as the private sector, non-governmental organisations (NGOs) and CHWs.
- Diabetes: Tight control of blood sugar at home and compliance to regular planned visits to CHC.
- Ultraviolet exposure: Use sunglasses and hats when there is prolonged exposure.
- Awareness campaigns: Eye care education delivered in homes, schools and other community settings. Integrate eye-health preservation in health promotion agendas carried out by the CHWs in the community.

4. **If you decide that visual loss is a priority in this community, what facility-based primary and secondary prevention interventions could you implement? (5 marks)**

*One mark for any of the five points given below.*

**Facility-based activities:**

- Improving accessibility to services to assess visual loss with the initiation of yearly screening days in partnership with optometrists, NGOs or ophthalmologists from the tertiary hospitals.
- Early detection of cataracts and a local standard operating procedure to do eye checks as part of routine examinations for all above the age of 50 years when presenting to the CHC with any complaints.
- Early diagnosis and control of people with diabetes. All diabetic patients should have a fundoscopy examination by trained personnel at least once a year.
- Continuing Professional Development (CPD) activity in the CHC to train staff on picking up and managing conditions that lead to visual loss.
- Regular measurement of data in the CHC, establishing a system of recording and monitoring the patients presenting with visual loss in the CHC.
- Consider patient-centred integrated eye care service in the CHC with emphasis on patient self-reporting: education pamphlets in the local languages that can be distributed to the patients on when/why to report to CHC with regard to visual loss.
- Engage with the clinic managers to make essential medicines and medical supplies available within health systems for eye care.

5. **Who could you approach as potential partners in addressing this disability burden for the community? (2 marks)**

*One mark for any of the two points given below*

- Regional hospital ophthalmological services, for example, ‘cataract blitzes’.
- Non-governmental organisations that would be able to provide corrective glasses for those operated, cataract trains and camps that mobilise services into the community.
- International humanitarian organisations, for example, Christoffel Blinden Mission.
- Media including social media can help spread awareness of the availability of services to the public.

**Further reading**

- Buso D, Reid S. Chapter 152: How to make a community diagnosis and prioritise health issues. In: Mash B, Blitz J, editors. South African family practice manual. 3rd ed. Pretoria: Van Schaik, 2015; p. 499–500.
- Marcus TS. Community orientated primary care, level 2. Topic 1, Unit 1. Pearson, 2013; p. 10.
- National guideline on the prevention of blindness in South Africa. Department of Health Directorate: Chronic Diseases, Disabilities and Geriatrics; 2002.
- World report on vision [homepage on the Internet]. Geneva: World Health Organization; 2019 [cited 2023 Jan 22]. Available from: https://www.who.int/publications/i/item/9789241516570
- Mash R, Gaede B, Hugo JJ. The contribution of family physicians and primary care doctors to community-orientated primary care. S Afr Fam Pract. 2021;63(1):1–5. https://doi.org/10.4102/safp.v63i1.5281

## Critical appraisal of quantitative research

Read the accompanying article carefully and then answer the following questions. As far as possible use your own words. Do not copy out chunks from the article. Be guided by the allocation of marks concerning the length of your responses.

- Tshivhase SE, Khoza LB, Tshitangano TG. Application of the information-motivation-behavioural skills model to strengthen eye care follow-up amongst glaucoma patients. Afr Vision Eye Health. 2021;80(1):8. https://doi.org/10.4102/aveh.v80i1.642

Total: 30 marks

1. Did the study address a focused question? Discuss. (2 marks)
2. Critically appraise the authors’ choice of study design to answer the research question. (4 marks)
3. Critically appraise the authors’ choice of the conceptual framework used to underpin the study’s design and data interpretation. (3 marks)
4. Critically appraise the sampling strategy. (5 marks)
5. Critically appraise how well the authors describe the data collection process. (4 marks)
6. Critically appraise the process followed to develop and validate the instrument used in this study. (3 marks)
7. Critically appraise the analysis and presentation of study data. (3 marks)
8. Use a structured approach (e.g. READER) to discuss the value of these findings to your practice. (6 marks)

Model answers

1. **Did the study address a focused question? Discuss. (2 marks)**
  In the abstract, the authors mentioned that they applied the information-motivation and behavioural skills model (IMBSM) in strengthening eye care follow-up among glaucoma patients in the Limpopo province of South Africa. In the introduction section, the authors expand on this aim by stating that they analysed a specific theoretical model for improving glaucoma patients’ knowledge, attitude and practices as a springboard for strategy development. Therefore, the study investigated how the application of the IMBSM can strengthen eye care follow-up in this patient group.
  One may conclude that the research question for this observational study is focused, as it describes the population, the risk factor(s), and the outcome of interest. The population studied is focused, as these are the glaucoma patients attending eye care services in Limpopo. They introduced a specific theoretical model or lens to understand adherence issues to follow-up (the risk factors, which may result in lapses in care continuity) and they had a certain outcome in mind, namely to strengthen eye care follow-up.
2. **Critically appraise the authors’ choice of study design to answer the research question. (4 marks)**
  The authors use a descriptive cross-sectional observational study to answer the research question.
  When considering the strength and appropriateness of this study design, one may conclude that this design is appropriate as it can assess the prevalence of an outcome of interest such as non-adherence to glaucoma medication, as well as the prevalence of any factors associated with it.
  Although a qualitative exploratory study could also be used, this would not provide insights into the prevalence of factors but provide rich data on aspects of non-adherence that may not have been anticipated.
  Given the extensive literature on adherence to chronic medication and the factors associated with it, the authors were justified to presume that most of the categories of factors contributing to non-adherence were considered.
3. **Critically appraise the authors’ choice of the conceptual framework used to underpin the study’s design and data interpretation. (3 marks)**
  A conceptual framework is a helpful way to illustrate the relationships between variables and concepts in a study and is often informed by the literature and any related hypotheses. In this study, the authors use the IMBSM model that is described and established in the literature to explain and connect the various categories of factors that contribute to adherence to chronic medication.
  The IMBSM model used in this study connects the prevention of non-adherence to three areas/categories, namely information and knowledge factors, motivation factors and behavioural skills factors. There are numerous models of understanding adherence, including the Health Belief Model or the World Health Organization’s Multidimensional Adherence Model. The latter model categorises patient-related factors, condition-related factors, social and economic factors, and therapy-related factors as dimensions of adherence.
  However, this study’s conceptual framework narrows the focus to knowledge, attitude and behavioural factors. While this may lead to the exclusion of other social, economic and health system factors, it is still consistent with the study’s overall aim of exploring areas of realistic intervention through information provision and behavioural modification. Nevertheless, the reader needs to be made aware of the complex mix of contextual factors that influence the individual’s ability to respond to ‘cues to action’; this should have been mentioned in the manuscript’s limitations section.
4. **Critically appraise the sampling strategy. (5 marks)**
  The authors employed a non-probability convenience sampling strategy and described the sample size of 450 as adequate for achieving reliable results. While the practicalities of being unable to randomly sample all patients with glaucoma on treatment in the district are understandably difficult, a systematic random sample could have still been attempted among the groups of patients approached, who attended the hospital for care and met the criteria.
  The hospital is the only ophthalmology referral centre in the entire region. This means that almost all public patients diagnosed with glaucoma would have been in the hospital’s care. Hospital records might have been used to ascertain the total number of patients with glaucoma to assist with calculating a representative sample size.
  It may have been useful if the authors included a clearer description in the study setting of how the local health system is organised and how this facilitates patient access to the services at Elim Hospital. A description of the distances between facilities and whether the services consist of only follow-up at the hospital or whether there is an outreach service, as these affect follow-up.
  Importantly, the authors do not describe in more replicable detail, how they calculated the sample size. Typically, in purely descriptive cross-sectional studies, a reference would be made to similar studies where a particular prevalence of the outcome in question (e.g. percentage of patients with poor adherence) would have been used to calculate their study’s sample size.
  It is also notable that the authors choose to exclude patients who were not attending consistently for 3 years. This means that patients who were very poorly adherent were not included in the study, thereby contributing to selection bias.
5. **Critically appraise how well the authors describe the data collection process. (4 marks)**
  The data collection process involved trained interviewers who provided the interviews. However, the authors indicate that many of the participants required assistance as they could not read or write and that the ‘researchers’ assisted them. It is not clear why the trained interviewers did not fulfil this role. Perhaps the authors implied that these interviewers were the researchers.
  Furthermore, it is also not clear how the interviewers were trained and supervised and how consent was taken. The professional background and language(s) of the interviewers were not described. Were these trained interviewers familiar with the community context and were they able to administer the instrument in the participants’ preferred or home language?
  Lastly, the authors do not describe where in the hospital the questionnaires were administered and whether confidentiality and anonymity were maintained or ensured.
6. **Critically appraise the process followed to develop and validate the instrument used in this study. (3 marks)**
  The study’s process for developing and validating the instrument is described in fair detail. They describe the establishment of face validity through their piloting of the questionnaire on 45 patients or participants. They also describe that they ensured alignment of the instrument questions with the objectives and the literature, which include alignment with the conceptual framework (content validity).
  However, they do not describe the details of how they established content validity. These could include the description of how many panel experts were used to review the instrument.
  The instrument was developed in the English language and subsequently translated into the ‘local language’. Neither the process of translation was described nor the local language was defined. Typically, the process of translation requires a series of forward and back translations, to ensure that the data collection instrument is aligned appropriately with the language and cultural context.
7. **Critically appraise the analysis and presentation of study data. (3 marks)**
  The study data are appropriately presented in tables using categories that are aligned with the conceptual framework. Participants’ responses are presented in proportions (percentages), which enables comparison.
  Unfortunately, not many inferences can be made from the findings as no inferential statistical analysis was conducted, nor were sample sizes calculated for such analyses. For example, no conclusions can be made about whether the patients with lower levels of knowledge of glaucoma demonstrated statistically significant lower levels of adherence.
  Some of the study data raise questions about the construct validity of the instrument. For example, it is not fully clear how the questions on patients’ beliefs about the cause of glaucoma revolved around only three possible answers (i.e. witchcraft, hereditary, and unknown).
8. **Use a structured approach (e.g. READER) to discuss the value of these findings to your practice. (6 marks)**

The READER format may be used to answer this question:

- Relevance to family medicine and primary care?
- Education – does it challenge existing knowledge or thinking?
- Applicability – are the results applicable to my practice?
- Discrimination – is the study scientifically valid enough?
- Evaluation – given the aforementioned, how would I score or evaluate the usefulness of this study to my practice?
- Reaction – what will I do with the study findings?

*The answer may be a subjective response but should be one that demonstrates a reflection on the possible changes within the student’s practice within the South African public healthcare system. It is acceptable for the student to suggest how their practice might change, within other scenarios after graduation (e.g. private general practice). The reflection on whether all important outcomes were considered is, therefore, dependent on the reader’s perspective (is there other information you would have liked to see?).*

*A model answer could be written from the perspective of the family physician employed in the South African district health system:*

- R: This study is relevant to the African primary care context because many patients with glaucoma are also followed up at primary care clinics and district hospitals while under the care of the regional hospital or tertiary ophthalmology service.
- E: While the challenge of adherence to chronic medication is not new, this study does reveal some local features that are arguably helpful to clinicians in this region. However, the challenges with representativity and the lack of more inferential statistical analysis limit what can be meaningfully drawn from the study.
- A: It is not possible to generalise the study’s findings to the wider South African setting, as the study was conducted in a specialist ophthalmology service using a non-probability sampling method.
- D: Regarding discrimination, the study has fundamental design flaws, bringing its validity into question. The lack of a description of how the sample size was calculated is problematic, which in turn affects what conclusions can be drawn even from the basic description of the statistical findings. Furthermore, those patients who are lost to follow-up are not represented at all and their reasons for non-adherence may be entirely different and unknown, which presents a selection bias to the findings that are not addressed. There is also an opportunity lost concerning applying inferential statistical methods.
- E: The topic and the findings are essential for informing any strategies for tailoring interventions that support adherence in this area and setting. Knowing the specific details of the major contributors to non-adherence to glaucoma medication in this group of patients is crucial. Given the design flaws in the study, this is not satisfactorily clear.
- R: Although the study has design and analytical flaws, the challenge of adherence to chronic medications, in general, is significant. These preliminary findings may be helpful for a follow-up explanatory study with a subset of these patients for richer details on adherence. More importantly, attempting to contact and follow-up with those who have not followed up with the service at all for such a study may reveal richer findings that could support intervention strategies for addressing and preventing non-adherence.

Total: 30 marks

**Further reading**

- Pather M. Evidence-based family medicine. In: Mash B, editor. Handbook of family medicine. 4th ed. Cape Town: Oxford University Press, 2017; p. 430–453.
- MacAuley D. READER: An acronym to aid critical reading by general practitioners. Br J Gen Pract. 1994;44(379):83–85.
- The Critical Appraisals Skills Programme (CASP). CASP checklists [homepage on the Internet]. 2023 [cited 2023 Feb 04]. Available from: https://casp-uk.net/casp-tools-checklists/
- Goodyear-Smith F, Mash B, editors. How to do primary care research. Boca Raton, FL: CRC Press, Taylor and Francis Group; 2019.

## Objectively structured clinical examination (OSCE) scenario

### Objective of station

This station tests the candidate’s ability to manage a patient with new onset unilateral visual loss.

### Type of station

Integrated consultation.

### Role player

Simulated patient: Adult male or female.

### Instructions to the candidate

- You are the family physician working at a district hospital. Please consult with this patient, who presents to the emergency unit.
- Your task: Please consult with this patient and develop a comprehensive management plan.
- You do not need to do an examination on this patient. All examination findings will be provided on request.

### Instructions for the examiner

- This is an integrated consultation station in which the candidate has 15 min.
- Familiarise yourself with the assessor guidelines, which detail the required responses expected from the candidate.
- No marks are allocated. In the mark sheet (Table 1), tick off one of the three responses for each of the competencies listed. Make sure you are clear on what the criteria are for judging a candidate’s competence in each area.
- Provide the following information to the candidate when requested: examination findings:
  ■ Please switch off your cell phone.
  ■ Please do not prompt the student.
  ■ Please ensure that the station remains tidy and is reset between candidates.

### Guidance for examiners regarding Figure 2

- The aim is to establish that the candidate has an effective and safe approach to managing acute persistent visual loss in an adult.
- Working definition of competent performance: The candidate effectively completes the task within the allotted time, in a manner that maintains patient safety, even though the execution may not be efficient and well structured:
  ■ *Not competent*: Patient safety is compromised (including ethical-legally) or the task is not completed.
  ■ *Competent*: The task is completed safely and effectively.
  ■ *Good*: In addition to displaying competence, the task is completed efficiently and in an empathic, patient-centred manner (acknowledges patient’s ideas, beliefs, expectations, concerns/fears).
- Establishes and maintains a good clinician–patient relationship.
  ■ The competent candidate is respectful and engages with the patient in a dignified manner.
  ■ The good candidate is empathic, compassionate and collaborative, facilitating patient participation in key areas of the consultation.
- Gathering information:
  ■ The competent candidate gathers sufficient information to establish a working diagnosis (*acute persistent visual loss secondary to acute angle-closure glaucoma*).
  ■ The good candidate additionally has a structured and holistic approach (*excludes other causes such as trauma, infection, foreign body, vascular insufficiency or neural; pays attention to pain and the accompanying emotional distress*).
- Clinical reasoning
  ■ The competent candidate identifies the diagnosis (*acute closed-angle glaucoma needing emergency care*) and acknowledges *the accompanying extreme distress*.
  ■ The good candidate makes a specific diagnosis (*acute closed-angle glaucoma*) and has a structured approach to addressing the patient’s illness experience (*analgesia; normalises distress; recognises immediacy of analgesic need).*
- Explaining and planning
  ■ The competent candidate uses clear language to explain the problem to the patient and uses strategies to ensure patient understanding (*questions OR feedback OR reverse summarising*).
  ■ The good candidate additionally ensures that the patient is actively involved in decision-making, paying particular attention to knowledge-sharing and empowerment, given the emergency of the situation and the further assessments needed to confirm the diagnosis.
- Management
  ■ The competent candidate proposes appropriate intervention (immediate discussion and referral to an ophthalmologist for complete assessment and rapid intervention).
  ■ The good candidate provides counselling to the patient on what she or he can expect at the ophthalmology department: Slit lamp or gonioscope examination; tomography. *Mentions a discussion with an ophthalmologist for possible administration of timolol and pilocarpine eyedrops and intravenous acetazolamide while awaiting transfer*.

**FIGURE 2:**
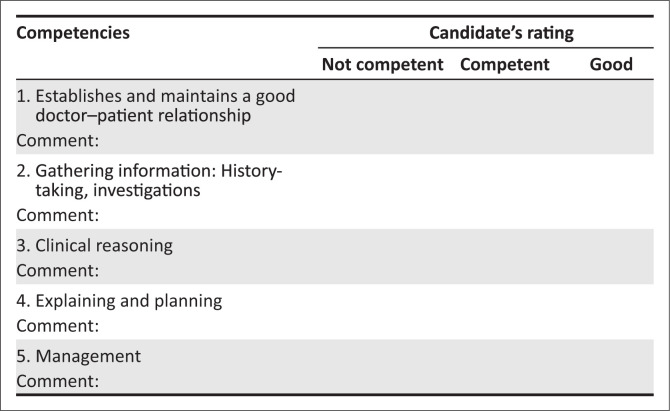
Marking sheet for objectively structured clinical examination station scenario.

### Role player instructions

#### Appearance and behaviour

Adult male or female, 40–60 years old, in pain and anxious.

**Opening statement:**

‘Doctor, I have had this terrible pain in my right eye since this morning, and now I can’t see properly … please help me … Am I going blind?’

#### History

- Open responses: Freely tell the doctor:
  ■ You have no idea why you have this problem. It started on its own yesterday and made you nauseous and is very painful. This is the first time ever that you have had this problem. You took paracetamol and codeine about 3 h ago, with no effect.
- Closed responses: Only tell the doctor if asked:
  ■ Vision: Very hazy vision and lights have halos around them.
  ■ The pain is the worst pain you’ve ever felt, with a throbbing deep behind your right eye into your head.
  ■ There is no family history of glaucoma.
  ■ You have no other medical problems.
  ■ You live a healthy lifestyle – eat well and exercise regularly.
  ■ You have been smoking since the age of 20 years – a pack lasts about 2 days.

**Your medical history**

- You have no medical issues that you know of.
- Ideas, concerns and expectations: You’re very worried that you will go blind and the pain is overwhelming.

### Examination findings

**Vital signs**

- Heart rate: 123/min. Easily palpable pulses.
- Blood pressure: 140/85 mmHg.
- Temperature: 36.5 °C.
- Random blood glucose level: 5.2 mmol/L.
- Point of care haemoglobin: 13.3 g/dL.

**Examination findings**

- Red, tearing right eye. Pupil not reacting to light. Cornea seems hazy and swollen. The globe feels harder than the right eye, and painful to palpation. Acuity: sees light, and hands at 30 cm only.
- Left eye, including visual acuity, is normal.
- All systemic examination reveals no abnormalities.

**Further reading**

- Leveque T. Approach to the person with acute persistent visual loss [homepage on the Internet]. UpToDate; 2021 [cited 2023 Feb 08]. Available from: https://www.uptodate.com/contents/approach-to-the-adult-with-acute-persistent-visual-loss?search=retinal%20detachment&source=search_result&selectedTitle=1~150&usage_type=default&display_rank=1
- Weizer JS. Angle-closure glaucoma [homepage on the Internet]. UpToDate; 2021 [cited 2023 Feb 08]. Available from: https://www.uptodate.com/contents/angle-closure-glaucoma?search=retinal%20detachment&topicRef=6902&source=see_link

